# Occipital and parietal non-invasive brain stimulation enhances perceptual learning and transfer: evidence from high-frequency tRNS

**DOI:** 10.3389/fnins.2026.1794676

**Published:** 2026-06-11

**Authors:** Yating Jin, Zhen Zhen, Rui Hua, Yue Ding, Yonghui Wang, Ya Li

**Affiliations:** 1 School of Psychology, Shaanxi Normal University, Xi’an, China; 2 Shaanxi Provincial Key Laboratory of Behavior and Cognitive Neuroscience, Shaanxi Normal University, Xi’an, China

**Keywords:** contour detection, learning transfer, occipital cortex, parietal cortex, perceptual learning, transcranial random noise stimulation

## Abstract

Perceptual training yields specific, long-lasting improvements, yet its transfer to untrained conditions is often limited. This tension has been proposed to involve interactions between early sensory plasticity and higher-order parietal processes, with the early visual cortex contributing to stimulus-specific learning and parietal regions potentially supporting more flexible generalization. However, causal evidence comparing how occipital and parietal stimulation modulates learning and transfer remains limited. To address this gap, we combined high-frequency transcranial random noise stimulation (tRNS) with a classical perceptual training paradigm. We examined whether occipital and parietal tRNS differentially enhance learning and whether they modulate transfer. Forty-one participants were randomly assigned to one of three groups: active tRNS over occipital cortex, active tRNS over parietal cortex, or sham stimulation, during multi-session training on a contour detection task. Results revealed that the learning dynamics exhibited distinct stage-specific modulations, with parietal tRNS accelerating early-stage acquisition and occipital tRNS sustaining learning rates during the later asymptotic phase. Furthermore, both active stimulations significantly increased the overall magnitude of learning gains compared to sham. Importantly, however, only parietal tRNS reliably enhanced transfer, particularly across curvature. These results provide causal evidence that occipital and parietal stimulation differentially modulate the behavioral dynamics of perceptual learning and transfer. This pattern is consistent with hierarchical accounts in which sensory and higher-order systems may contribute differently to learning stability and flexibility, while also highlighting the potential of targeted neuromodulation to enhance distinct phases and outcomes of perceptual learning.

## 1 Introduction

Visual perceptual learning refers to long-lasting improvements in perceptual performance resulting from repeated practice on a visual task (Goldstone, 1998). It serves as a powerful model for studying experience-dependent plasticity in the adult brain (Gilbert and Li, 2012; Shibata et al., 2014). A persistent puzzle in this field is the tension between specificity (Furmanski et al., 2004; Schoups et al., 2001), where improvements are confined to the trained stimulus and transfer (Roberts and Carrasco, 2022; Watanabe and Sasaki, 2015), where benefits generalize to untrained conditions. Identifying the mechanisms that determine this balance between specificity and transfer is critical, not only for theory but also for leveraging perceptual learning in applied domains such as clinical rehabilitation.

A central theoretical debate revolves around the primary locus of this plasticity underlying these behavioral outcomes. One dominant view, the representation change account, posits that learning refines sensory representations in the visual cortex (e.g., Fahle et al., 1995; Yang and Maunsell, 2004). This view is supported by physiological evidence, such as single-unit recordings in awake macaques, showing training-induced sharpening of neuronal orientation tuning in V1 (Schoups et al., 2001), to enhanced location-specific population responses revealed by human fMRI studies (Furmanski et al., 2004), or refining population codes in early visual cortex (Yan et al., 2014), along with corroborative human neuroimaging and electrophysiological findings (Bao et al., 2010; Jehee et al., 2012; Schwartz et al., 2002). In contrast, the influential Integrated Reweighting Theory (IRT) proposes that learning primarily optimizes the readout of sensory information by higher-order decision-related areas, a mechanism that could support learning and transfer (Dosher et al., 2013; Dosher and Lu, 1999; Lu and Dosher, 2022). Parietal areas, including the intraparietal sulcus, are critical hubs for perceptual decision formation, supporting evidence accumulation and the transformation of sensory inputs into decision evidence (Bollimunta and Ditterich, 2012; Gold and Shadlen, 2007; Zhang et al., 2022; Zhou and Freedman, 2019), and may also contribute to feedforward reweighting operations that assign decision weights to sensory inputs during learning. Converging evidence indicates that training is accompanied by changes in frontoparietal regions implicated in decision-related processing and attentional gating of sensory evidence. For example, human functional magnetic resonance imaging (fMRI) studies reveal that learning-induced decreases in blood-oxygen-level-dependent (BOLD) signals within frontoparietal attention networks correlate with behavioral improvement (Mukai et al., 2007), and multivariate pattern analyses demonstrate that higher-order frontal regions track the updating of decision variables during perceptual learning (Kahnt et al., 2011). And also, electrophysiological work shows that training enhances the ability of decision-related parietal neurons to decode task-relevant information (Law and Gold, 2008). Given the extensive evidence supporting both views, rather than viewing these cortical regions in strict opposition, a critical question emerges regarding the specific causal contributions of early sensory (e.g., occipital) versus higher-order (e.g., parietal) regions to the acquisition and subsequent generalization of perceptual learning?

Although previous studies have consistently implicated both occipital and parietal cortices in perceptual learning, they leave two critical causal questions unresolved. First, despite robust correlational links between learning and neural changes in occipital (Mukai et al., 2007; Zhang and Kourtzi, 2010) and parietal (Chen et al., 2015; Law and Gold, 2008) regions, it remains unknown whether their activity is causally necessary for driving the learning process itself. Second, and more importantly, direct evidence is lacking on how these areas differentially support the transfer of learning. Theoretical models, such as the IRT, posit that perceptual learning refers to the experience-driven optimization of connection weights from representational layers to decision units, emphasizing changes in the mapping from representations to decision processes (Dosher et al., 2013; Lu and Dosher, 2022). They propose that higher-order areas (e.g., parietal) are crucial for generalization, whereas early sensory areas (e.g., occipital) may underpin specificity. To test these causal relationships, previous studies have elegantly employed transcranial magnetic stimulation (TMS) as a “virtual lesion.” For instance, Chang et al. (2014) used rTMS to reveal that perceptual training shifts the neural locus of generalization from the posterior parietal cortex to the lateral occipital cortex after learning. Similarly, Jia et al. (2021) applied TMS during test phases to demonstrate the causal involvement of the parietal cortex in maintaining post-training working memory representations. However, because these studies applied stimulation primarily to assess offline performance constraints before or after learning, it remains unclear whether modulating these regions during the acquisition phase would differentially shape the formation of learning and transfer.

Transcranial random noise stimulation (tRNS) offers a unique methodological solution to address this gap. By non-invasively modulating cortical excitability and promoting a state conducive to experience-dependent plasticity (Chaieb et al., 2015; Potok et al., 2021, 2022), tRNS allows for targeted, causal intervention during the training phase itself. Crucially, applying tRNS to different cortical areas provides a direct test of the competing theoretical frameworks. Prior studies have shown that occipital tRNS boosts visual perceptual learning (Fertonani et al., 2011; He et al., 2023), consistent with the representation change accounts emphasizing early sensory plasticity. In contrast, while the IRT posits that higher-order parietal regions optimize sensory readout to facilitate generalization (Dosher et al., 2013), the efficacy of parietal tRNS in visual transfer has not been directly tested, despite promising indirect evidence from fMRI (Jia et al., 2020) and cognitive training studies (Cappelletti et al., 2013).

To address this precise knowledge gap, the present study applied online high-frequency tRNS (hf-tRNS) over either the occipital or parietal cortex throughout a multi-session contour training paradigm. This design allows us to extend the established parieto-occipital framework from identifying offline performance states to dynamically probing how these networks causally construct learning and generalization. Specifically, we asked two questions: (1) does occipital or parietal tRNS enhance perceptual learning relative to sham control? and (2) do these stimulations differentially modulate the transfer of learning to untrained stimulus conditions? Drawing on the aforementioned theoretical frameworks, we tested two distinct hypotheses. First, following the representation change account, occipital tRNS should primarily enhance learning gains for the trained stimulus by refining early sensory processing. Second, in line with the IRT, parietal tRNS should optimize reweighting, thereby promoting both the learning and the transfer to untrained stimuli. By comparing these stimulation effects within a single design, we aim to provide direct behavioral evidence on how modulating distinct cortical hierarchies selectively shapes the stability and flexibility of visual perceptual learning.

## 2 Methods

### 2.1 Ethics and participants

To ensure adequate statistical power, an *a priori* power analysis was conducted using G*Power 3.1 (Faul et al., 2007). Assuming a medium effect size (Cohen’s *f* = 0.25, corresponding to $\eta_{p}^{2}$ = 0.059), an alpha level of 0.05, a desired power of 0.80, and a moderate correlation among repeated measures (*r* = 0.50), the analysis indicated that a minimum total sample size of 36 participants was required to detect a significant within-between interaction. The final sample of 41 participants thus exceeded this requirement.

Forty-three participants were recruited and randomly assigned to three groups (Parietal, Occipital, Sham). Data from forty-one participants were retained for analysis (7 males; age: *M* = 20.61, SD = 1.72 years; Parietal: *n* = 15, 12 females, age *M* = 20.20, SD = 1.32, range = 19–22 years; Occipital: *n* = 13, 10 females, age *M* = 20.69, *SD* = 1.80, range = 19–25 years; Sham: *n* = 13, 12 females, age *M* = 20.77, SD = 1.86, range = 18–25 years). All participants were right-handed, had normal or corrected-to-normal vision, and reported no color blindness. Participants had no prior experience with contour detection tasks and were unaware of the specific experimental hypotheses. Exclusion criteria included metallic implants, a history of epilepsy or seizures, neurological or psychiatric disorders, current use of psychiatric medication, a history of craniotomy, pregnancy, and participation in any electrical or magnetic brain stimulation experiments within the past 4 weeks. Two participants were excluded for failing to meet the predefined learning criterion, defined as having a mean accuracy below 70% in the trained orthogonal-straight condition at jitter levels of 0° and 10° during post-test. The study protocols were approved by the Ethics Committee on Human Experimentation of Shaanxi Normal University (Approval Number: 2025-09-02) following the Declaration of Helsinki. Written informed consent was obtained from the participants before the experiments.

### 2.2 Experimental equipment

The experiment was conducted using a 27-inch In-Plane Switching (IPS) display (2,560 × 1,440 resolution, 100 Hz refresh rate). Participants were seated in a darkened room at a viewing distance of 67 cm, with their heads stabilized by a chin rest. Stimulus presentation was programmed in MATLAB R2017b (The MathWorks, Inc., Natick, MA, United States) with PsychToolbox-3 (Brainard, 1997), and visual stimuli were generated using the Grouping Elements Rendering toolbox (Demeyer and Machilsen, 2012).

### 2.3 Experimental design and process

The present study adopted a 3 (Stimulation group: Parietal, Occipital, Sham) × 2 (Orientation: Trained, Untrained) × 2 (Contour Regularity: Orthogonal, Collinear) mixed design, with contour regularity and orientation as within-subject factors and stimulation as a between-subject factor. Given that contour-integration learning is often stimulus-dependent and can show strong specificity under task-driven training (Li et al., 2008; Zhang and Kourtzi, 2010), we explicitly designed the task to evaluate transfer along two separable curvature and orientation dimensions. Curvature is a key geometric factor constraining contour detectability (Khuu et al., 2017), motivating an explicit test of curvature-based transfer. The untrained orientation was strictly operationalized as the orthogonal counterpart to a participant’s assigned training orientation (i.e., 135° if trained at 45°, and vice versa). Collinear contours refer to stimuli in which local elements (i.e., Gabor elements) are aligned with the global contour path, whereas orthogonal contours refer to stimuli in which local elements are oriented perpendicularly (90°) relative to the global contour path. Notably, to accommodate the aforementioned test of cross-curvature transfer, orthogonal contours included two subtypes (orthogonal-straight and orthogonal-curved), whereas collinear contours included only a straight subtype (collinear-straight) as a baseline condition.

**Figure 1:**
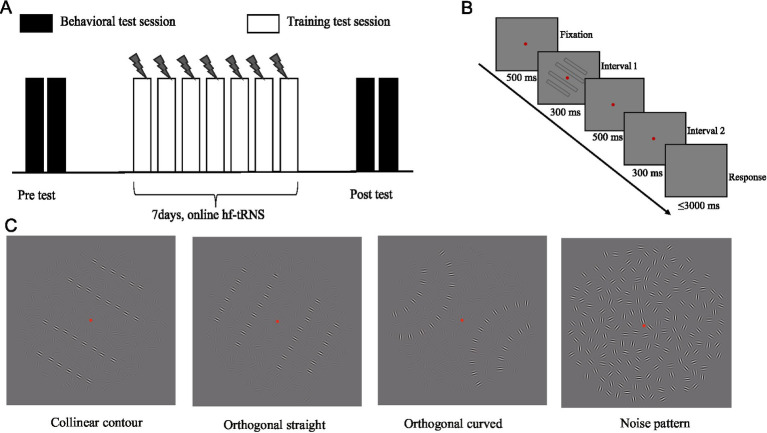
Experimental design, task, and stimuli for the contour detection experiment. **(A)** Overall procedure. Participants completed a pre-test phase consisting of two test sessions administered on separate days, followed by seven consecutive daily training sessions, and then a post-test phase consisting of two test sessions administered on separate days. During each training session, HF-tRNS was applied online according to group assignment. Note: Although the pre-test and post-test phases were each split across two consecutive days to prevent fatigue, data from these split sessions were pooled to yield a single pre-test and post-test measurement. **(B)** Trial sequence in the two-interval forced-choice (2IFC) task. Each trial comprised two stimulus intervals; one contained a contour embedded in noise and the other contained a noise-only pattern. Participants indicated which interval contained the contour. **(C)** Example stimuli. Contours were formed by Gabor elements embedded in a field of randomly oriented background elements. In collinear contours, local Gabor orientations were aligned with the global contour path, whereas in orthogonal contours they were perpendicular to the contour path; noise-only patterns contained background elements without a contour. For illustrative purposes, the contour elements in these examples are depicted with higher contrast than the background to enhance visibility. During the actual experiment, all Gabor elements were presented at identical contrast.

The experiment comprised a pre-test, seven daily training sessions (approximately 30 min per day), and a post-test (approximately 60 min). During the training phase, different tRNS stimulations were applied to the three participant groups (see Figure 1A). In all phases, observers performed a two-interval forced choice (2AFC) contour detection task. Learning and transfer were quantified using threshold-based indices (learning rate, mean improvement, and transfer index), which are formally defined in Section 2.5.

#### 2.3.1 Procedure and stimuli

Each trial began with a red fixation point presented for 500 ms, followed by the first interval stimulus displayed for 300 ms. After a 500 ms inter-stimulus interval, the second interval stimulus was presented for 300 ms. One interval contained a contour stimulus, and the other contained a noise stimulus. The order of target and noise intervals was randomized and counterbalanced across trials, such that the contour appeared equally often in the first and second intervals. Participants indicated which interval contained the contour stimulus. If the contour appeared in the first interval, they pressed the “F” key; if it appeared in the second interval, they pressed the “J” key. This response mapping was fixed across participants. Responses were permitted only after both intervals had been presented. The maximum response time was 3,000 ms. Each trial ended upon response or timeout, followed by a 500 ms inter-trial interval. Throughout each trial, the red fixation point remained continuously visible at the center of the screen, and participants were instructed to maintain fixation (see Figure 1B).

Stimuli were presented within a circular aperture with a radius of 4.1° of visual angle. On target intervals, the display contained four contour segments embedded in a background of randomly positioned and oriented Gabor elements. The contour segments were defined by aligned Gabor elements: two inner segments comprising 8 elements each and two outer segments comprising 6 elements each, 28 contour elements in total. The four contour segments were presented at four fixed locations symmetrically arranged around fixation (two inner and two outer positions) along the global contour orientation. To discourage local density cues, adjacent elements were separated by a minimum center-to-center spacing of 0.42°, and a Voronoi-based Monte Carlo local-density test was applied, only nonsignificant displays were retained. Under this spacing constraint, the number of background elements varied (*M* = 187.25, SD = 3.54, range = 173–202), yielding an average total of 215.25 elements per display (SD = 3.54, range = 201–230). Gabor elements were cosine gratings modulated by a Gaussian envelope with spatial frequency 8.3 cycles per degree, envelope SD *σ* = 0.08°, and contrast 100%.

Global orientation was anchored at 45° or 135° and sampled within ±15° in 1.5° increments, excluding the 0° offset, with trial order randomized. Task difficulty was manipulated by independently applying orientation jitter to each contour element relative to the mean contour orientation; jitter values were sampled from a Gaussian distribution (*M* = 0, SD = jitter magnitude). During the test phase, jitter magnitudes were set to 0°, 10°, 20°, 30°, and 45°. In the training phase, jitter was adjusted adaptively based on individual performance.

Noise stimulus pattern was created by shuffling the orientations of all Gabor elements while preserving their spatial positions. Thus, each noise pattern shared identical element placement with the contour stimuli but lacked coherent contour structure. Examples of both contour and noise patterns are shown in Figure 1C.

#### 2.3.2 Training session

During training, participants were trained on the orthogonal-straight contour condition at their assigned trained orientation. Half of the participants were trained with contours oriented at 45°, while the other half were trained with contours oriented at 135°. Consequently, the unassigned global orientation served as the explicitly defined untrained condition for that participant during test phases. Task difficulty was manipulated by adjusting the magnitude of orientation jitter; larger jitter values produced less coherent contours and therefore increased task difficulty.

A three-down-one-up adaptive staircase procedure (converging to approximately 79.4% accuracy) was used to estimate each participant’s contour detection threshold during training. Under this rule, the jitter magnitude increased by 1° after three consecutive correct responses and decreased by 1° after a single incorrect response, starting from an initial jitter of 0°. Each participant completed nine staircases per day. Each staircase terminated after 15 reversals or after reaching 100 trials. The threshold for each staircase was defined as the average of the last eight reversals, and the daily threshold was computed as the mean across the nine staircases. The starting jitter for the first staircase of each training day was set to the threshold obtained from the final staircase of the previous day. Each daily training session lasted approximately 30 min, including self-paced breaks between staircases. Auditory feedback was provided for incorrect responses during training.

#### 2.3.3 Test session

During the pre-test and post-test phases, contour detection performance was assessed across all within-subject conditions defined by Orientation (Trained, Untrained) and Contour Regularity (Orthogonal, Collinear). To probe cross-curvature transfer, the orthogonal condition was further subdivided at test into orthogonal-straight and orthogonal-curved contours. Accordingly, the test phase included six conditions formed by crossing Orientation (Trained, Untrained) with three contour types (Collinear-straight, Orthogonal-straight, Orthogonal-curved); each condition was tested under five jitter levels (0°, 10°, 20°, 30°, and 45°), with trials mixed and randomized.

In total, each test phase comprised 1,200 trials, organized into 30 blocks of 40 trials. To minimize cognitive fatigue and visual adaptation and thereby maintain stable psychophysical performance, each test phase was split into two sessions administered on consecutive days, with 15 blocks completed per day. Although conducted on separate days, these two sessions were treated as a single assessment both conceptually and analytically. Accordingly, data from the two sessions were combined to derive one pre-test threshold and one post-test threshold for each participant. The total duration of each test phase was approximately 60 min. No feedback was provided during the test phase. Unlike the adaptive staircase used during training, test performance was assessed using the method of constant stimuli; details of threshold estimation are described in Section 2.5.

#### 2.3.4 Practice session

Before the pre-test, all participants completed a short practice block consisting of 40 trials to familiarize themselves with the task procedure. Practice trials were drawn from the same stimulus set as the formal test, including 3 (Contour Type: Collinear-straight, Orthogonal-straight, Orthogonal-curved) × 2 (Orientation: Trained, Untrained) × 5 (Jitter: 0°, 10°, 20°, 30°, and 45°), presented in a mixed and randomized order. During this practice session, auditory feedback was provided immediately after incorrect responses to facilitate rule learning; no feedback was provided for correct responses.

### 2.4 Transcranial random noise stimulation

**Figure 2:**
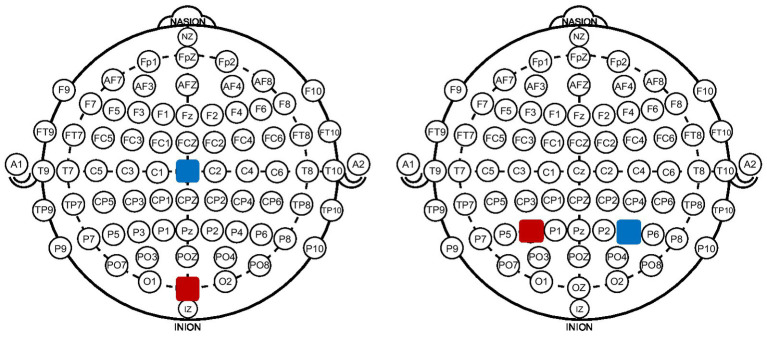
Schematic of electrode placement. Left is stimulation electrodes were positioned over the occipital cortex (Oz) and the vertex (Cz). Right is stimulation electrodes were positioned over the parietal cortex (P3 and P4). The electrode positions were defined by the International 10–20 EEG system.

High-frequency transcranial random noise stimulation (hf-tRNS; 101–640 Hz) with 0 mA DC offset was applied during training. Stimulation was delivered using 5 × 5 cm rubber electrodes enclosed in saline-soaked sponge pads using a battery-driven constant-current stimulator. Electrode positions were determined using the International 10–20 EEG system and were kept fixed within each group throughout the experiment. For the parietal stimulation group, electrodes were placed over P3 and P4; for the occipital stimulation group, electrodes were placed over Oz and Cz (see Figure 2). These specific montages were selected based on established computational electric field modeling from prior literature. Previous modeling studies using conventional macroscopic sponge electrodes (e.g., 25–35 cm^2^) have consistently demonstrated that the Oz-Cz montage effectively concentrates current density over the early visual cortex (Datta et al., 2009; Neuling et al., 2012). Furthermore, recent electric field simulations of hf-tRNS have explicitly confirmed that a bilateral P3-P4 montage induces localized peak electric field strengths over the posterior parietal cortex, specifically targeting the intraparietal sulcus (Contò et al., 2021). Electrode-skin impedance was maintained below 20 kΩ before stimulation. Sham participants were randomly assigned to either the occipital or parietal montage, with equal numbers in each subgroup. For the active stimulation groups, hf-tRNS was delivered for 20 min at 1.0 mA peak-to-peak, including a brief ramp-up at stimulation onset and followed by a 20 s ramp-down. For sham stimulation, all settings matched the active protocol, but the current was ramped down and terminated after 20 s to mimic the initial sensation of stimulation.

### 2.5 Data analysis

During the pre-test and post-test phases, contour detection thresholds were estimated for each participant across all test conditions. Thresholds were obtained using the method of constant stimuli and psychometric-function fitting implemented in the *psignifit* toolbox in MATLAB (Schütt et al., 2016). Psychometric fits were performed on proportion-correct data, and threshold estimates were extracted from the fitted functions. Specifically, for each condition, accuracy was computed at each jitter level, and a psychometric function was fitted; the jitter magnitude corresponding to 70% correct was taken as the detection threshold. To quantify learning effects, we derived three threshold-based indices: learning rate, mean improvement (MI), and transfer index (TI).

#### 2.5.1 Training session

To capture performance change during training, learning rate was assessed with session-level log-linear function and block-level piecewise linear spline regression. The former was used to replicate previous studies to make our results comparable across studies. Furthermore, building on prior studies using segmented regression for learning trajectories (Levi et al., 1997; Zhou et al., 2012), we conducted a block-level piecewise linear spline regression to capture the temporally heterogeneous phases of perceptual learning, which typically consist of an initial rapid improvement followed by a plateau (Censor et al., 2012; Shibata et al., 2014). This finer-grained (i.e., block-level analysis) modeling prevents session-level averages from obscuring critical temporal dynamics (Cochrane et al., 2024; Yang et al., 2025).

##### 2.5.1.1 Session-level analysis

The session-level learning rate was estimated by fitting a log-linear function, y=a+b·log(x) , to each participant’s thresholds for the trained orthogonal-straight condition across seven training days only, where *y* denotes the threshold (jitter magnitude) and *x* is the training-day index (*x* = 1 to 7 for Training Days 1–7). The slope *b* was taken as the learning rate.

##### 2.5.1.2 Block-level spline regression

For the finer-grained analysis, block order across the training sequence was log_10_-transformed to account for non-linear learning rates, and a spline term was introduced after a predefined knot to estimate slope changes. To preserve within-session continuity, the knot was restricted to the first block of a training day. It was operationally placed where performance reached approximately 90% of the final improvement, capturing the transition to the asymptotic phase while retaining sufficient room to observe post-knot changes. The model was specified as:


$$\left\{ \begin{array}{cc} \beta_{0}+(\beta_{1}+\beta_{2}k)\log_{10}(i)+\varepsilon , & i<t\\ \beta_{0}+(\beta_{1}+\beta_{2}k)\log_{10}(i)+(\beta_{3}+\beta_{4}k)\log_{10}(\frac{i}{t})+\varepsilon , & i\geq t \end{array}\right.$$

The model estimated block-by-block thresholds using the Sham group as the reference. Because pre-test thresholds did not differ significantly across groups, interactions between group and the intercept were excluded from the model. The primary parameters of interest included the reference group’s early-stage learning slope ( $\beta_{1}$ ) and post-knot slope change ( $\beta_{3}$ ), alongside group-specific interaction terms ( $\beta_{2}$ , $\beta_{4}$ ) to evaluate how active stimulation adjusted both the early learning rate and the later-stage trajectory. Because preliminary analyses confirmed no significant group differences in pre-test thresholds (see Supplementary file S1), we constrained the model to a single shared intercept ( $\beta_{0}$ ). This model was used to test whether (1) the Sham group showed significant learning during the early phase of training, (2) the overall learning slope decreased after the knot, and (3) the Occipital and Parietal groups differed from the Sham group in early learning rate or in later-stage changes in learning trajectory. Parameters were estimated using ordinary least squares regression.

#### 2.5.2 Test session

##### 2.5.2.1 Mean improvement

Mean improvement quantified overall learning gain and was computed as $\mathrm{\Delta Th}={\mathrm{Th}}_{\text{post}}-{\mathrm{Th}}_{\mathrm{pre}}$ for the trained condition, where ${\mathrm{Th}}_{\text{post}}$ and ${\mathrm{Th}}_{\mathrm{pre}}$ denote the post-test and pre-test thresholds, respectively; because larger jitter thresholds indicate better performance, larger ΔTh values reflect greater learning gains.

##### 2.5.2.2 Transfer index

To quantify transfer relative to learning on the trained task, we defined a Transfer Index based on pre- to post-test improvements. It is designed to quantify the extent to which training gains generalize to untrained conditions, relative to the gains observed in the trained condition. Specifically, TI is calculated as the ratio of pre-post improvement in the untrained condition to the improvement in the trained condition:


$$\mathrm{TI}=\frac{\text{Post}\_\text{Test}-\mathrm{Pre}\_\text{Test}(\text{Untrained Condition})}{\text{Post}\_\text{Test}-\mathrm{Pre}\_\text{Test}(\text{Trained Condition})}$$

Following this definition, we derived two specific indices, (1) Cross-orientation transfer: ΔTh (untrained orientation, orthogonal-straight)/ΔTh (trained orientation, orthogonal-straight): (2) cross-curvature transfer: ΔTh (trained orientation, orthogonal-curved)/ΔTh (trained orientation, orthogonal-straight).

#### 2.5.3 Statistical analysis

Prior to the primary analyses, assumption checks were systematically conducted. No outliers exceeding three standard deviations from the condition mean were identified. Normality of the data distributions was assessed and confirmed using the Shapiro–Wilk test. For all ANOVAs, the assumption of sphericity was evaluated using Mauchly’s test. When sphericity was violated, Greenhouse–Geisser corrections were applied to the degrees of freedom, and corrected *p*-values are reported.

To test stimulation effects on the session-level learning rate, group differences were tested using separate one-way ANOVAs on learning rate (*b*), with Stimulation group (Parietal, Occipital, Sham) as the between-subjects factor. The significance of parameters was assessed using *t*-tests based on asymptotic standard errors. For each estimated parameter, the *t*-value and its corresponding two-tailed *p*-value are reported. To test stimulation effects on learning gains, we performed a 3 (Stimulation group: Parietal, Occipital, Sham) × 2 (Contour condition: Trained, Baseline control) mixed-design repeated-measures ANOVA on MI. The untrained collinear-straight contour (averaged across the trained and untrained orientations) was used as a within-group baseline control condition to evaluate possible improvements due to generic practice or test–retest effects. This specific collinear contour was chosen because previous studies have demonstrated that perceptual learning is highly specific to the trained contour type. Finally, to test stimulation effects on transferability across curvature and orientation, group differences were tested using separate one-way ANOVAs on TI, with Stimulation group (Parietal, Occipital, Sham) as the between-subjects factor. Significant omnibus effects were followed up with Bonferroni-corrected pairwise comparisons. All statistical analyses were performed using RStudio.

## 3 Results

To evaluate group comparability, we further examined demographic balance across groups. Age did not differ significantly among the three groups, *F* (2, 38) = 0.59, *p* = 0.561, and the gender distribution was also comparable across groups (Fisher’s exact test, *p* = 0.669).

### 3.1 Training dynamics

#### 3.1.1 Session-level results

**Figure 3:**
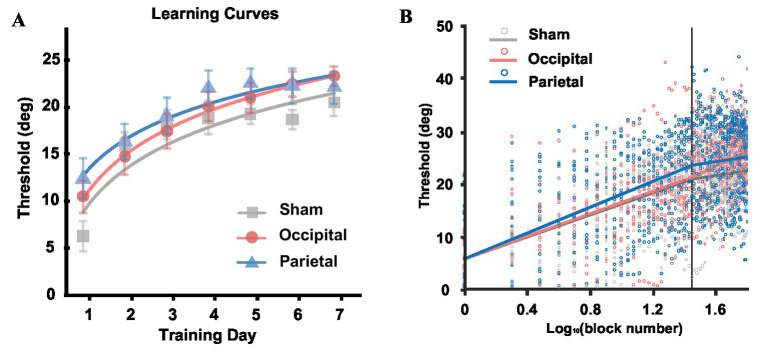
Learning trajectories during training at session and block levels. **(A)** Group-averaged detection thresholds (jitter magnitude) at the trained orientation from pre-test through the seven training days. Points show group means ± SEM, and solid lines show log-linear fits to the group-averaged trajectory. **(B)** Block-level threshold trajectories during training as a function of log-transformed block number. Each dot represents the threshold from a single training block, and solid lines represent fitted spline regression curves for the Sham, Occipital, and Parietal groups. Dashed vertical lines indicate block boundaries across the seven training days, and the thicker dashed line indicates the predefined knot at Block 28.

To examine potential differences in learning rate across stimulation conditions, a one-way ANOVA with Stimulation group (Parietal, Occipital, Sham) as a between-subjects factor was conducted on the learning rate derived from the log-linear fits to the training thresholds in the trained orthogonal-straight condition (see Figure 3A). The analysis revealed no significant main effect of stimulation group, *F* (2, 38) = 0.68, *p* = 0.515, *η_p_*^2^ = 0.034, indicating that the rate of improvement across training sessions did not significantly differ among the three groups. Additional analyses including age and gender as covariates were reported in the Supplementary material (see S3.1) and yielded the same overall pattern.

#### 3.1.2 Block-level results

To further characterize the temporally heterogeneous phases of training beyond the session-level average, we conducted a supplementary block-level spline regression analysis of block-wise threshold data (see Figure 3B). As detailed in the Supplementary material (see S2), Block 28 was selected as the predefined knot because it provided a central transition point—where performance had approached asymptote (approx. 90% of final improvement)—while retaining sufficient data on both sides for stable slope estimations.

Consistent with our model specification, the shared intercept ( $\beta_{0}$ ) was significant, *b* = 5.34, SE = 0.60, *t* (2576) = 8.93, *p* < 0.001, which represented the common initial performance level. For the reference group (Sham), the early-stage learning slope ( $\beta_{1}$ ) was significantly positive, *b* = 10.61, SE = 0.55, *t* (2576) = 19.18, *p* < 0.001, confirming robust early acquisition. Furthermore, the post-knot slope change ( $\beta_{3}$ ) for the reference group was significantly negative, *b* = −6.00, SE = 2.47, *t* (2576) = −2.43, *p* = 0.015. This demonstrates that the learning rate of the sham group flattened significantly after Block 28, successfully capturing the transition from initial rapid improvement to a later plateau-like phase.

Regarding group differences in these stage-specific dynamics, the interaction between the occipital group and the early-stage term was not significant, *b* = 0.23, SE = 0.38, *t* (2576) = 0.59, *p* = 0.558. In contrast, the interaction between the parietal group and the early-learning term ( $\beta_{2}$ ) was significantly positive, *b* = 1.82, SE = 0.37, *t* (2576) = 4.90, *p* < 0.001, indicating that parietal stimulation significantly accelerated the early-stage learning rate relative to the Sham group.

For later-stage changes, the interaction between the parietal group and the post-knot slope change ( $\beta_{4}$ ) was not significant, *b* = −1.79, SE = 3.16, *t* (2576) = −0.57, *p* = 0.572. Conversely, the interaction between the occipital group and the post-knot slope change was significantly positive, *b* = 9.41, SE = 3.27, *t* (2576) = 2.88, *p* = 0.004. This positive interaction indicates that the post-knot reduction in learning slope was significantly attenuated in the occipital group relative to the sham group, reflecting a less pronounced slowing of learning rate during the later stage of training.

To evaluate the robustness of the results, we also examined the results with knots placed at *t* = 19 and *t* = 37. The observed pattern was consistent with that obtained using the primary knot at *t* = 28, confirming the robustness and reliability of the spline regression analysis. Overall, this block-level spline analysis provides quantitative evidence that the learning trajectories diverged across groups during training. Specifically, the two active stimulations exerted distinct, stage-specific modulations on the online acquisition dynamics: parietal tRNS accelerated the early-stage learning rate, whereas occipital tRNS sustained the learning process by attenuating the plateau effect during the later stage.

### 3.2 Learning gains and transfer

#### 3.2.1 Mean improvement

**Figure 4:**
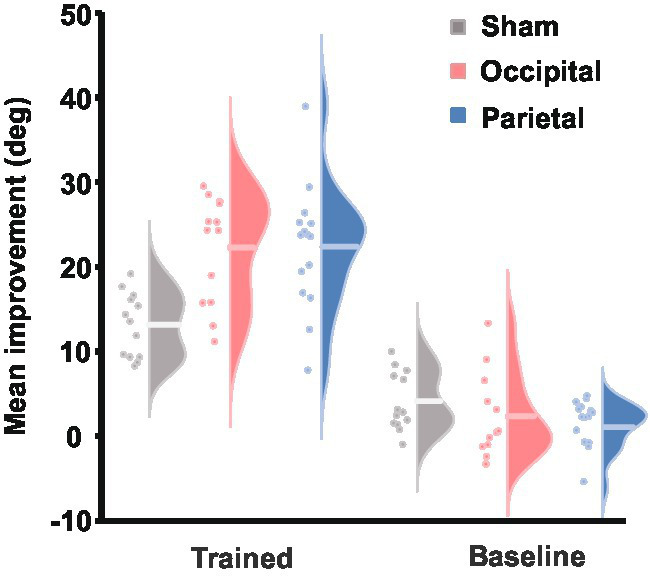
Mean improvement across stimulation groups. Mean improvement (calculated as post-test minus pre-test thresholds) is shown separately for the trained (orthogonal-straight) and the baseline control condition, defined as the untrained collinear-straight contour averaged across the trained and untrained orientations. Half-violin plots show the distribution of participant values, with individual participants overlaid as dots and horizontal white lines indicating group means. Positive values indicate improved performance (higher tolerated jitter). Gray, orange, and blue denote the Sham (*n* = 13), Occipital (*n* = 13), and Parietal (*n* = 15) groups, respectively.

To test stimulation effects on learning gains, we performed a 3 (Stimulation group: Parietal, Occipital, Sham) × 2 (Contour condition: Trained, Baseline) mixed-design ANOVA on mean improvement (see Figure 4). The analysis revealed a significant main effect of stimulation group, *F* (2, 38) = 4.83, *p* = 0.014, *η_p_*^2^ = 0.203, indicating that mean improvement differed across stimulation groups. There was also a significant main effect of contour condition, *F* (1, 38) = 163.50, *p* < 0.001, *η_p_*^2^ = 0.811, with greater improvement in the trained condition than in the baseline condition. Importantly, the stimulation group × contour condition interaction was significant, *F* (2, 38) = 8.44, *p* < 0.001, *η_p_*^2^ = 0.308, suggesting that the effect of stimulation differed between the trained and baseline conditions.

Bonferroni-corrected simple effects analyses were conducted within each contour condition. For the trained condition, both active stimulation groups showed greater mean improvement than the sham group (Occipital vs. Sham: *t* (38) = 3.66, *p* = 0.002, Cohen’s *d* = 1.19; Parietal vs. Sham: *t* (38) = 3.75, *p* = 0.002, Cohen’s *d* = 1.22). No significant difference was observed between the occipital and parietal groups in the trained condition (*t* (38) = 0.04, *p* = 1.00, Cohen’s *d* = 0.01). In contrast, no significant group differences emerged in the baseline condition (all *p* ≥ 0.149). To further ensure that the pooling of orientations did not bias these results, we performed sensitivity analyses using alternative baseline definitions (separately for trained and untrained orientations), which confirmed that the main pattern of results remained robust (see Supplementary material S4). Additional analyses including age and gender as covariates were reported in the Supplementary material (see S3.2) and confirmed that the main pattern of results remained unchanged.

#### 3.2.2 Transfer index

**Figure 5:**
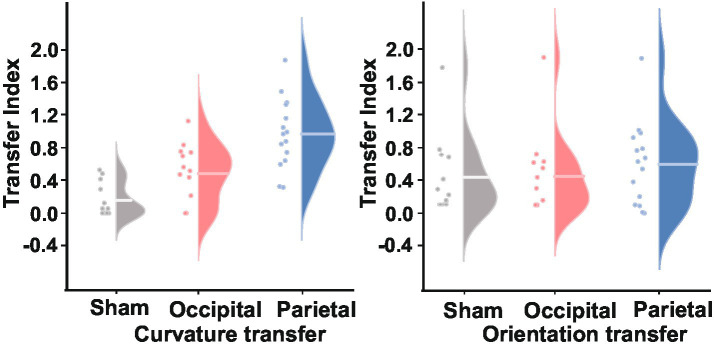
Transfer indices across stimulation groups. Half-violin plots display the distribution of transfer indices for each group, with individual participants shown as dots and horizontal lines indicating group means. Cross-curvature transfer is shown on the left and cross-orientation transfer on the right. Gray, orange, and blue denote the Sham (*n* = 13), Occipital (*n* = 13), and Parietal (*n* = 15) groups, respectively.

To examine whether Stimulation group (Parietal, Occipital, Sham) influenced transfer performance, we conducted separate one-way ANOVAs on the two transfer indices, with stimulation group as a between-subjects factor (see Figure 5). For cross-orientation transfer, the main effect of stimulation group was not significant, *F* (2, 38) = 0.50, *p* = 0.609, *η_p_*^2^ = 0.026, suggesting that stimulation group did not affect cross-orientation transfer. For cross-curvature transfer, a significant main effect of stimulation group was observed, *F* (2, 38) = 20.21, *p* < 0.001, *η_p_*^2^ = 0.515. *Post hoc* pairwise comparisons with Bonferroni corrections showed that the parietal group exhibited a significantly higher transfer index than both the occipital group (*t* (38) = 3.79, *p* = 0.002, Cohen’s *d* = 1.23) and the sham group (*t* (38) = 6.27, *p* < 0.001, Cohen’s *d* = 2.03). The difference between the occipital and sham groups was not significant (*t* (38) = 2.39, *p* = 0.066, Cohen’s *d* = 0.78). Additional analyses including age and gender as covariates were reported in the Supplementary material (see S3.3) and confirmed that the group effect on cross-curvature transfer remained robust. A further analysis examining the orientation specificity of the cross-curvature transfer effect was reported in the Supplementary material (see S5), which suggested that the parietal transfer advantage was primarily observed at the trained orientation.

## 4 Discussion

This study combined a classical perceptual learning paradigm with hf-tRNS to elucidate the distinct roles of the parietal and occipital cortices. Consistent with our first hypothesis, both the occipital and parietal tRNS groups exhibited significantly greater overall improvement than the sham group. Furthermore, the learning dynamics exhibited distinct stage-specific modulations, with parietal tRNS accelerating early-stage acquisition learning rate and occipital tRNS sustaining learning rates during the later asymptotic phase. These findings indicate that both visual and parietal cortical regions play causal roles in perceptual learning. More importantly, and as predicted by our second hypothesis, we observed a clear dissociation in transfer outcomes. Only parietal tRNS reliably enhanced transfer, particularly across curvature. This result provides causal evidence that the parietal and occipital cortices make differential contributions to the flexibility of learning.

By applying hf-tRNS during training, we provide causal evidence for the functional involvement of occipital and parietal regions in perceptual learning, thereby addressing a key limitation of correlational studies. Previous neurophysiological and neuroimaging studies have shown training-induced changes in these cortical regions (Chen et al., 2015; Mukai et al., 2007; Schoups et al., 2001; Zhang and Kourtzi, 2010). However, in those studies, neural activity was measured directly or indirectly before and after training, but not during the training sessions. Consequently, it remained unclear whether the observed neural changes caused improvements in perceptual abilities or were merely a consequence of them. In the present study, we applied hf-tRNS to the occipital or parietal cortices during training and observed enhanced learning effects, providing direct evidence for their causal functional roles. Specifically, the enhancement of learning reflected in two aspects: the online, stage-specific modulation of learning dynamics (the acquisition process) and the offline increase in the overall magnitude of gains (the final performance outcome). While overall improvement reflects the cumulative result of training, the observed stage-dependent shifts in learning rates—parietal acceleration in the early stage and occipital maintenance in the late stage—demonstrate that tRNS fundamentally reshapes the trajectory of acquisition.

Crucially, this online interventional approach explicitly extends the framework established by TMS studies (e.g., Chang et al., 2014; Jia et al., 2021). While prior work utilized offline virtual lesions to map how the neural locus of generalization shifts after learning is complete, our study uncovers how modulating neural excitability during active acquisition fundamentally alters the post-learning outcomes. Our findings provide new evidence that the parietal cortex is not merely a transient processing stage supplanted after training. Rather, its active engagement during training facilitates both immediate learning dynamics and subsequent offline consolidation and transfer. We thus demonstrate that occipital plasticity primarily supports feature-specific improvements, whereas concurrent parietal plasticity is indispensable for both robust overall learning and broader generalization.

The present findings also help clarify how the proposed mechanisms of hf-tRNS may be expressed behaviorally during perceptual learning. Because hf-tRNS does not target frequency-specific oscillatory activity, the observed learning and transfer effects are unlikely to reflect entrainment. Instead, they likely reflect online effects of tRNS—such as an increased signal-to-noise ratio for weak inputs during stimulation—as well as tRNS-induced after-effects associated with increased cortical excitability and more efficient neural signal transmission and processing within neuronal populations (Potok et al., 2022; van der Groen et al., 2022). In the context of perceptual learning, these mechanisms may facilitate the processing of task-relevant sensory information during training and support the formation of learning-related plastic changes. Whether these changes remain specific to the trained condition or generalize to untrained conditions may depend on the functional contribution of the stimulated region. Occipital stimulation likely modulates encoding or refinement of sensory representations, whereas parietal stimulation may preferentially influence the selection, readout, or weighting of information that enables flexible transfer.

Previous studies have shown that occipital tRNS boosts perceptual learning in discrimination tasks involving orientation (Fertonani et al., 2011; He et al., 2023; Pirulli et al., 2013) or motion stimuli (Herpich et al., 2019). Consistent with these findings, we observed enhanced learning in a contour detection task, which requires the spatial integration of local elements into a global pattern. This boosting effect of occipital tRNS aligns with extensive previous work and can be interpreted through dual theoretical lenses. According to the representation change account, occipital tRNS may have facilitated learning by enhancing the neural representation of the trained contour stimulus, possibly via increased cortical excitability and plasticity (Fertonani et al., 2011; Terney et al., 2008). Indeed, previous studies suggested that occipital cortices play a critical role in the representation of contour stimuli (Gilbert and Li, 2012; Kuai et al., 2017; Li et al., 2008; Li et al., 2019; Yan et al., 2014). Thus, occipital tRNS in our study may have amplified contour representation in visual areas, leading to improved integration and enhanced learning. Supporting this, previous research has shown that occipital tRNS enhances global motion detection performance, a task that requires integrating visual information across time (Herpich et al., 2019). Alternatively, within the IRT framework, occipital stimulation might have optimized evidence accumulation (van der Groen et al., 2018), thereby strengthening the reweighting of sensory signals to decision units. Under this account, occipital tRNS likely strengthens the training-driven reweighting of neural signals from visual areas to higher brain regions, ultimately improving learning. Our results from occipital stimulation are consistent with both accounts and do not differentiate between them.

Critically, our novel finding that parietal tRNS boosts perceptual learning is broadly consistent with the IRT’s core premise that higher-order areas involved in decision formation are integral to the learning process itself. Previous studies have found that perceptual training alters neural activity in the parietal cortex (Law and Gold, 2008) and its connection with sensory areas (Chen et al., 2015; Jia et al., 2020). Parietal tRNS may enhance learning by optimizing the readout of sensory evidence from sensory areas, potentially through increasing the excitability or plasticity of neural populations responsible for these reweighting computations. Under this account, parietal tRNS may have facilitated a more efficient extraction of task-relevant signals from the visual cortex and more selectively assigning decision weights to informative sensory channels, thereby amplifying the impact of training. The effect of parietal tRNS on transfer is also parsimoniously explained by the IRT. According to this theory, generalization emerges when training refines connection weights not only from feature-specific representations in early visual cortex but also from feature-invariant representations in higher-order visual regions (Lu and Dosher, 2022). In this framework, the degree of specificity or transfer depends on the overlap of connection patterns shared between the trained and transfer stimuli. Our finding that transfer was specific to curvature, but not orientation aligns with this view. On the one hand, electrophysiological evidence shows that macaque V4 neurons tuned to the straight segments also respond to low-curvature contours (Nandy et al., 2013), suggesting that these stimuli share invariant feature representations. Optimizing the readout weights from such shared representations would naturally support the transfer across curvature observed here. On the other hand, electrophysiological and fMRI studies also report orientation selectivity for contour representations (Chen et al., 2014; Kuai et al., 2017). It is possible that parietal tRNS improved transfer across curvature while maintaining specificity across orientations by simultaneously optimizing the readout of both invariant and orientation-selective representations.

Another possibility is that parietal tRNS enhances perceptual learning by improving attentional processes. A growing body of evidence indicates that attention not only enhances the efficiency of learning but also determines its capacity for generalization by regulating the communication between early sensory and higher-order cortical areas (Roelfsema et al., 2010; Szpiro and Carrasco, 2015; Xiong et al., 2016). Indeed, several tRNS studies have shown that parietal stimulation modulates attention, both at the behavioral level and within attention networks (Contò et al., 2021; Harty and Kadosh, 2019; Shalev et al., 2018). Perceptual training studies further support the critical role of attention, finding that learning is accompanied by changes in attentional regions and in their coupling with the visual cortex, which correlate with the amount of learning (Lewis et al., 2009; Mukai et al., 2007). In our study, parietal tRNS may have enhanced learning through strengthened attention networks. Furthermore, research on perceptual learning indicates that attention facilitates transfer across feature and location (Donovan et al., 2020; Nguyen et al., 2020; Roberts and Carrasco, 2022). It is thus plausible that by increasing top-down attention, parietal tRNS could optimize its functional state and thereby improve the transfer. However, if enhanced attention were the primary or sole mechanism driving transfer, we would expect it to facilitate transfer across both curvature and orientation dimensions. Our finding of transfer specific to curvature, but not orientation, suggests that a generalized attention enhancement cannot fully account for the observed pattern of results.

It is also possible that parietal tRNS enhanced learning by increasing the strength or effectiveness of top-down feedback signals sent from parietal regions to the early visual cortex. This account is also compatible with the observed transfer effect in our study. These feedback signals can function as a “gain controller,” dynamically shaping sensory input by amplifying task-relevant information and suppressing noise (Cohen and Maunsell, 2009; Poort et al., 2022; Reynolds and Chelazzi, 2004). Under the feedback change framework, perceptual learning has been proposed to involve strengthened top-down feedback connections from higher cognitive control regions (such as the parietal cortex) to early sensory areas (Seitz, 2020). Consistent with this view, recent causal evidence demonstrates that corticocortical feedback from higher-order visual regions is essential for shaping figure-ground segregation and contextual integration in early visual cortex (Xin et al., 2025).

From a broader perspective, our parietal findings may be tentatively interpreted in terms of two complementary mechanisms: a predominantly feedforward reweighting mechanism or a feedback optimization process. Although these accounts differ in where they place the primary learning-induced change in information flow, they are not mutually exclusive (Maniglia and Seitz, 2018; Seitz, 2020). Parietal tRNS may, on the one hand, directly accelerate the computation of feedforward weight selection, and on the other, enhance the efficacy of this region as a source of top-down feedback signals to the sensory cortex. The stage-dependent modulation effect is broadly consistent with the possibility that the facilitatory contribution of parietal stimulation was expressed relatively early in training, which is compatible with accounts emphasizing early selection, weighting, or readout of task-relevant sensory information. A similar hybrid interpretation may also apply to the occipital stimulation results. It is plausible that refined sensory representations and accelerated evidence accumulation jointly contribute to the reweighting of information passed to decision units, and that these processes may operate in parallel. Under this view, occipital tRNS would not only modulate sensory representations but also possibly optimize their downstream weighting to enhance learning.

It is also important to consider whether the transfer effect observed in the parietal tRNS group could partly reflect oculomotor or attentional sampling strategies in addition to perceptual mechanisms, since the parietal cortex is known to contribute to saccade planning, attentional selection and visual exploration (Huddleston et al., 2021; Ptak and Müri, 2013). Although the stimulus duration in our study does not strictly preclude eye movements, the behavioral pattern makes a completely nonspecific oculomotor account less likely. If parietal tRNS had primarily improved generic eye-movement control or fixation stability, such benefits would generalize broadly across all transfer conditions. Instead, the transfer advantage emerged exclusively for curved contours at the trained orientation. However, this pattern does not rule out more selective oculomotor or attentional sampling accounts. Recent evidence suggests that eye-movement-related learning and implicit oculomotor sampling can be highly feature- or task-specific (Desbordes et al., 2025; Grzeczkowski et al., 2023), indicating that the absence of broad generalization alone is not sufficient to exclude oculomotor contributions. This possibility is particularly relevant given that parietal tRNS accelerated early-stage acquisition, a phase during which participants may develop more efficient task-specific sampling or visual exploration strategies. Because eye movements were not recorded, the present data cannot determine whether parietal stimulation altered fixation stability, saccade latency, landing position, microsaccades, or task-specific exploration patterns. It is possible that the observed curvature transfer might result from a combination of perceptual, attentional, and oculomotor factors. Future studies combining neuromodulation with eye tracking, fixation-contingent trial exclusion, or gaze-contingent stimulus control will be needed to clarify the relative contributions of perceptual, attentional, and oculomotor mechanisms to learning transfer.

While previous studies reported that occipital tRNS promotes broader transfer in clinical populations, such as those with mild myopia and amblyopia (Camilleri et al., 2014; Campana et al., 2014), our study found that occipital stimulation in healthy observers enhanced learning without producing reliable transfer to untrained conditions. This discrepancy likely stems from differences in baseline visual function. In healthy systems, occipital tRNS may primarily consolidate the processing of the specifically trained stimuli. This interpretation is consistent with findings from healthy adults showing that occipital tRNS can facilitate perceptual learning without necessarily promoting broader generalization (Contemori et al., 2019; Liu et al., 2023).

Beyond these effects on learning and transfer, a noteworthy pattern emerged. The performance decline observed in the Sham group during the post-test was absent in the active tRNS groups. This decline was likely driven by the shift from a highly predictable training context (single, fixed condition) to a high-uncertainty post-test context (multi-condition, intermixed design), which is known to disrupt acquired performance advantages (Adini et al., 2004). Our findings align with this phenomenon. Crucially, the active tRNS groups did not exhibit a comparable post-test decline. One possible explanation for this stability is that online hf-tRNS applied during training made the learning outcomes more resilient to the contextual uncertainty of the post-test. This interpretation aligns with previous findings showing that tRNS effects are particularly evident under conditions with high contextual variability and low trial predictability (Popescu et al., 2016; Yao et al., 2021), and that tRNS can promote longer-lasting, stable plastic changes (van der Groen et al., 2022). Thus, tRNS may foster a robust form of learning less susceptible to shifts in task context. It would be interesting for further studies to directly test this possibility.

While tRNS provides causal evidence for the involvement of the stimulated areas, its effects are not perfectly focal and may influence interconnected networks. This limits our ability to pinpoint the exact neural populations or network-level changes responsible for the observed behavioral effects. In addition, the mechanistic interpretations regarding reweighting versus feedback, while grounded in theory, remain speculative based on the current behavioral and stimulation data alone. Another limitation is that eye movements were not recorded. Although the behavioral pattern argues against a completely nonspecific scanning account, it cannot exclude more selective oculomotor or attentional sampling strategies. Future studies combining tRNS with eye-tracking and high-resolution neuroimaging techniques will be essential for determining the relative contribution and interaction of these mechanisms. Such approaches will provide a more comprehensive understanding of the respective roles of parietal and occipital cortices in perceptual learning and generalization and directly test the hypotheses generated here.

## 5 Conclusion

In summary, our study shows that both occipital and parietal tRNS significantly increased the overall magnitude of perceptual learning, but exerted distinct, stage-specific effects on learning dynamics. Importantly, only parietal stimulation reliably promoted the transfer of learning gains. These findings provide causal evidence that occipital and parietal stimulation differentially modulate the behavioral dynamics of perceptual learning and transfer. More broadly, this pattern is consistent with hierarchical accounts in which early sensory and higher-order parietal systems may contribute differently to learning acquisition, stability, and generalization. This dissociation in stimulation effects points to hf-tRNS as a promising tool for enhancing the efficiency and flexibility of perceptual learning.

## Data Availability

All data, analysis, and task codes have been made publicly available on the Open Science Framework and can be accessed at https://osf.io/bs2gt/.
