# Supplementary material for: Occipital and parietal non-invasive brain stimulation enhances perceptual learning and transfer: evidence from high-frequency tRNS
Source: Front Neurosci. 2026 Jun 11;20:1794676. doi: 10.3389/fnins.2026.1794676 (PMC13294261; doi:10.3389/fnins.2026.1794676)
Supplement: Supplementary file 1 [file Data_sheet_1.docx]

Supplementary Material

**Occipital and Parietal Non-invasive Brain Stimulation Enhances Perceptual Learning and Transfer: Evidence from High-Frequency tRNS**

**Yating Jin^1, 2^** **^†^, Zhen Zhen^1, 2^** **^†^, Rui Hua^1, 2^, Yue Ding^1, 2^, Yonghui Wang^1, 2^*, Ya Li^1, 2^***

^1^ School of Psychology, Shaanxi Normal University, Xi’an 710062, China

^2^ Shaanxi Provincial Key Laboratory of Behavior & Cognitive Neuroscience, Shaanxi Normal University, Xi’an 710062, China

**Supplementary Result**

# S1 Pre-test Thresholds for the Trained Condition

To confirm that the three Stimulation groups did not differ in pretest performance, we conducted a one-way ANOVA on pre-test thresholds for the trained condition, with Stimulation group (Parietal, Occipital, Sham) as a between-subjects factor. The analysis revealed no significant main effect of group, *F*(2, 38) = 1.87, *p* = .169, *η*_p_²= .089. This result indicates that there were no significant differences among the three groups at pretest, providing a basis for excluding the group–intercept interactions term from the spline regression model.

# S2 Knot Selection

The knot was identified using a 90% improvement threshold. Earlier candidate knots fell within the steep phase of learning; later candidate knots occurred near asymptote, offering limited opportunity to detect post‑knot change (see Table S1).

Table S1. Candidate knots and corresponding threshold values.

| Block number | Mean threshold | Proportion of final improvement | Increase from previous candidate knot |
| --- | --- | --- | --- |
| 1 | 6.46 | 26.3% | 26.3% |
| 10 | 15.72 | 64.0% | 37.7% |
| 19 | 19.60 | 79.8% | 15.8% |
| 28 | 22.72 | 92.5% | 12.7% |
| 37 | 23.62 | 96.1% | 3.6% |
| 46 | 23.60 | 96.1% | 0.0% |
| 55 | 24.45 | 99.5% | 3.4% |

Supplementary Table S1 summarizes the candidate knots and their corresponding threshold values. Candidate knots were restricted to the first block of each training day to preserve within-session continuity. The mean threshold was calculated across the three stimulation groups at each candidate knot. The proportion of final improvement was calculated as:

$Pi=\frac{{Th}_{i}}{{Th}_{63}} \times100\%$,

where *Th_i_* denotes the threshold at the *i*th block and *Th_63_* denotes the threshold at the final training block. The fourth column shows the increase in this proportion relative to the previous candidate knot.

# S3 Effects of Age and Gender Covariates

# S3.1 Session-level Result

To examine whether the observed pattern of learning-rate results was influenced by demographic variables, we conducted a supplementary analysis including age and gender as covariates in the model. Learning rate was defined as the slope parameter (*b*) derived from log-linear fits to individual training thresholds across Days 1–7.

A one-way ANOVA including Stimulation group (Parietal, Occipital, Sham), Age, and Gender as predictors revealed no significant main effect of stimulation group, *F*(2, 36) = 0.80, *p* = .455, η*_p_*² = .043. Importantly, neither age, *F*(1, 36) = 0.75, *p* = .394, *η*_p_² = .020, nor gender, *F*(1, 36) = 0.73, *p* = .398, *η*_p_² = .020, showed significant effects on learning rate. Thus, the non-significant group effect on learning rate remained unchanged after controlling for age and gender.

# S3.2 Mean Improvement

To examine whether the observed effects on mean improvement were influenced by demographic variables, we conducted a supplementary mixed-design ANOVA including age and gender as covariates. The model included stimulation group (Parietal, Occipital, Sham) as a between-subjects factor and contour condition (Trained, Baseline) as a within-subjects factor.

The main effect of Stimulation group remained significant after controlling for age and gender, *F*(2, 36) = 3.97, *p* = .028, *η*_p_² = .181. The Stimulation group × Contour condition interaction also remained significant, *F*(2, 36) = 6.93, *p* = .003, *η*_p_² = .278, indicating that the differential effect of stimulation across contour conditions was preserved. Neither the main effect of age, *F*(1, 36) = 0.35, *p* = .556, *η*_p_² = .010, nor gender, *F*(1, 36) = 0.22, *p* = .642, *η*_p_² = .006, was significant. Interactions involving the covariates, including age × contour condition and gender × contour condition, were also not significant, both *ps* > .54. Follow-up comparisons showed the same pattern as the primary analysis: both active stimulation groups showed greater improvement than the Sham group in the trained condition, whereas no reliable group differences were observed in the baseline condition.

# S3.3 Transfer Index

To examine whether the observed effects on transfer performance were influenced by demographic variables, we conducted supplementary analyses including age and gender as covariates. Transfer indices were analyzed separately for cross-curvature transfer and cross-orientation transfer using one-way ANOVAs with Stimulation group (Parietal, Occipital, Sham) as a factor and age and gender as covariates.

For cross-curvature transfer, the main effect of stimulation group remained significant after controlling for age and gender, *F*(2, 36) = 19.96, *p* < .001, *η*_p_² = .526. Neither age, *F*(1, 36) = 0.31, *p* = .582, *η*_p_² = .008, nor gender, *F*(1, 36) = 1.06, *p* = .310, *η*_p_² = .029, showed significant effects. Follow-up comparisons showed the same pattern as the primary analysis, with greater cross-curvature transfer in the parietal group than in both the occipital and sham groups. For cross-orientation transfer, the main effect of stimulation group remained non-significant after controlling for age and gender, *F*(2, 36) = 0.57, *p* = .569, *η*_p_² = .031.Overall, these results indicate that the main transfer pattern was robust: stimulation group significantly modulated cross-curvature transfer, whereas no reliable group effect was observed for cross-orientation transfer after controlling for age and gender.

# S4 Sensitivity Analyses of Stimulation Effects Across Different Baseline Definitions

To verify that the pooling of orientations did not affect the core conclusions regarding learning gains, we conducted two sensitivity analyses using refined baseline definitions. A 3 (stimulation group: Parietal, Occipital, Sham) × 2 (contour condition: trained, baseline) mixed-design ANOVA was conducted. In the first analysis, the baseline was defined as the untrained collinear-straight contour at the trained orientation only. The analysis revealed a marginally significant main effect of stimulation group, *F*(2, 38) = 2.65, *p* = .084, *η*_p_² = .122, and a significant main effect of contour condition, *F*(1, 38) = 137.45, *p* < .001, *η*_p_² = .783. Importantly, the group × contour condition interaction was significant, *F*(2, 38) = 8.28, *p* = .001, *η*_p_² = .303. Bonferroni-corrected simple-effects analyses showed that in the trained condition, both active stimulation groups showed significantly greater mean improvement than the sham group (Occipital vs. Sham: *t*(38) = 3.66, *p* = .002, Cohen’s *d* = 1.19; Parietal vs. Sham: *t*(38) = 3.75, *p* = .002, Cohen’s *d* = 1.22), whereas no significant difference was observed between the occipital and parietal groups, *t*(38) = 0.04, *p* = 1.00, Cohen’s *d* = 0.01. In the baseline condition, no group differences were significant (all *p*s ＞ .151). Within-group comparisons confirmed that mean improvement was significantly greater for the trained contours than for the baseline contours across all groups (all *p*s ＜ .002, Cohen’s *d* ＞ 0.54).

In the second analysis, the baseline was redefined as the untrained collinear-straight contour at the untrained orientation only. The ANOVA revealed a significant main effect of stimulation group, *F*(2, 38) = 5.26, *p* = .010, *η*_p_² = .217, and a significant main effect of contour condition, *F*(1, 38) = 147.54, *p* < .001, *η*_p_² = .795. The group × contour condition interaction was also significant, *F*(2, 38) = 6.42, *p* = .004, *η*_p_² = .253. Bonferroni-corrected simple-effects analyses were conducted to further explore this interaction. Within the trained condition, both active stimulation groups exhibited significantly greater mean improvement compared to the sham group (Occipital vs. Sham: *t*(38) = 3.66, *p* = .002, Cohen’s *d* = 1.19; Parietal vs. Sham: *t*(38) = 3.75, *p* = .002, Cohen’s *d* = 1.22). No significant difference was observed between the occipital and parietal groups (*t*(38) = 0.04, p = 1.00, Cohen’s *d* = 0.01). Within the baseline condition, no significant differences emerged between any of the groups (all *p*s ＞ .921). Comparisons within each group showed that mean improvement for trained contours was significantly greater than for the baseline contours at the untrained orientation (all *p*s < .001, Cohen’s *d* ＞ 0.65). Collectively, these results indicate that the observed facilitatory effects of both occipital and parietal tRNS on learning gains are robust and independent of the specific baseline definition used.

# S5 Orientation Specificity of Cross-Curvature Transfer

To further examine whether the cross-curvature transfer effect was specific to the trained orientation, we compared curvature transfer indices between the trained-orientation and untrained-orientation orthogonal-curved conditions. A 3 (Stimulation group: Parietal, Occipital, Sham) × 2 (Orientation: Trained, Untrained) mixed-design ANOVA was conducted.

The analysis revealed a significant main effect of orientation, *F*(1, 38) = 26.43, *p* < .001, *η*_p_² = .410, indicating that the curvature transfer index at the trained orientation was higher than untrained-orientation orthogonal-curved condition, *t*(38) = 5.14, *p* < .001, Cohen’s *d* = 0.80. The main effect of stimulation group was also significant, *F*(2, 38) = 14.26, *p* < .001, *η*_p_² = .429. Bonferroni-corrected post hoc comparisons showed that the parietal group had a significantly higher transfer index than both the occipital group, *t*(38) = 2.71, *p* = .020, Cohen’s *d* = 1.09, and the Sham group, *t*(38) = 5.32, *p* < .001, Cohen’s *d* = 2.02, whereas the difference between the occipital and sham groups was not significant, *p* = .070.

Importantly, the group × orientation interaction was significant, *F*(2, 38) = 5.68, *p* = .007, *η*_p_² = .230. The simple effects analyses showed that, at the trained orientation, the parietal group had significantly greater curvature transfer than both the occipital group, *t*(38) = 3.80, *p* = .002, Cohen’s *d* = 1.44, and the sham group, *t*(38) = 6.28, *p* < .001, Cohen’s *d* = 2.38. In contrast, at the untrained orientation, no group differences were significant after Bonferroni correction (all *ps* ≥ .266). This pattern indicates that the cross-curvature transfer advantage in the parietal group was specific to the trained orientation.
